# NFATc4 Regulates *Sox9* Gene Expression in Acinar Cell Plasticity and Pancreatic Cancer Initiation

**DOI:** 10.1155/2016/5272498

**Published:** 2015-11-30

**Authors:** Elisabeth Hessmann, Jin-San Zhang, Nai-Ming Chen, Marie Hasselluhn, Geou-Yarh Liou, Peter Storz, Volker Ellenrieder, Daniel D. Billadeau, Alexander Koenig

**Affiliations:** ^1^Department of Gastroenterology and Gastrointestinal Oncology, University Medical Center Göttingen, Robert-Koch Street 40, 37075 Göttingen, Germany; ^2^Schulze Center for Novel Therapeutics, Division of Oncology Research, Mayo Clinic, 200 1st Street SW No. W4, Rochester, MN 55905, USA; ^3^School of Pharmaceutical Sciences and Key Laboratory of Biotechnology and Pharmaceutical Engineering, Wenzhou Medical University, Wenzhou, Zhejiang, China; ^4^Department of Cancer Biology, Mayo Clinic Cancer Center, 4500 San Pablo Road, Jacksonville, FL 32224, USA

## Abstract

Acinar transdifferentiation toward a duct-like phenotype constitutes the defining response of acinar cells to external stress signals and is considered to be the initial step in pancreatic carcinogenesis. Despite the requirement for oncogenic Kras in pancreatic cancer (PDAC) development, oncogenic Kras is not sufficient to drive pancreatic carcinogenesis beyond the level of premalignancy. Instead, secondary events, such as inflammation-induced signaling activation of the epidermal growth factor (EGFR) or induction of Sox9 expression, are required for tumor formation. Herein, we aimed to dissect the mechanism that links EGFR signaling to Sox9 gene expression during acinar-to-ductal metaplasia in pancreatic tissue adaptation and PDAC initiation. We show that the inflammatory transcription factor NFATc4 is highly induced and localizes in the nucleus in response to inflammation-induced EGFR signaling. Moreover, we demonstrate that NFATc4 drives acinar-to-ductal conversion and PDAC initiation through direct transcriptional induction of *Sox9*. Therefore, strategies designed to disrupt NFATc4 induction might be beneficial in the prevention or therapy of PDAC.

## 1. Introduction

Pancreatic ductal adenocarcinoma (PDAC) is among the most aggressive human malignancies with a 5-year survival rate of less than 7% [1]. PDAC arises from well-defined precursor lesions, with pancreatic intraepithelial neoplasia (PanIN) being the best described [2, 3]. Oncogenic mutations of the* Kras* gene represent the defining molecular alteration in pancreatic carcinogenesis and can be detected in early PanIN lesions and in more than 90% of advanced human PDAC, leading to the current dogma that this genetic event is required for PDAC initiation and progression [4, 5]. Lineage-tracing studies have demonstrated that acinar cells expressing oncogenic Kras lose their grape-like structure and undergo a de- and transdifferentiation process termed acinar-to-ductal metaplasia (ADM) to generate metaplastic lesions with a duct-like phenotype [4, 5]. Characteristically, dedifferentiation of mature exocrine pancreatic cells involves a gene expression profile that highly resembles the one found in the embryonic pancreas [6, 7], including activation of Notch signaling or induction of the sex-determining region Y-box 9 (Sox9) transcription factor [6, 8]. Importantly, the progenitor-like characteristics of metaplastic acinar cells make them more susceptible to Kras-induced oncogenesis [6, 9]. In fact, oncogenic Kras hijacks the acinar redifferentiation process that occurs in regenerative pancreatic tissue and instead promotes a transition from metaplastic cells to PanIN precursor lesions that eventually progress to invasive PDAC [10, 11].

Significantly, ADM formation and neoplastic progression in the context of Kras mutation occur with a low penetrance and a long latency, unless secondary events arise which drive pancreatic carcinogenesis beyond the stage of premalignancy [2, 3, 5]. Inflammatory environmental cues are well appreciated to promote pancreatic carcinogenesis on the background of oncogenic Kras mutations [5], thus reflecting epidemiologic studies characterizing chronic pancreatitis as the major risk factor for PDAC development [12]. Examination of chronic pancreatitis patient samples revealed an upregulation of epidermal growth factor receptor expression in metaplastic pancreatic lesions [13–15]. Interestingly, transgenic EGFR ligand overexpression promoted the formation of pancreatic metaplasia [16, 17], whereas EGFR inactivation utilizing genetic or pharmacological approaches suppressed acinar-to-ductal transdifferentiation by oncogenic Kras activation and inflammation [9, 18]. Taken together, these data suggest that EGFR activation is required for inflammation-driven acinar dedifferentiation and PDAC initiation. Nevertheless, the exact molecular mechanisms that link EGFR activation to acinar transdifferentiation remain elusive.

Herein we sought to determine how inflammatory signaling pathways in metaplastic pancreatic cells bridge EGFR activation to transcriptional induction of key mediators of acinar cell dedifferentiation. In particular, we sought to characterize the impact of NFATc4 (nuclear factor of activated T-cells 4) on transcriptional activation of the ductal fate determinant* Sox9* during acinar-ductal transdifferentiation. We show that NFATc4 gene expression is highly induced during EGFR-stimulated acinar-to-ductal conversion in acinar explants and is required for duct formation and the expression of Sox9 in this* in vitro* system. Moreover, we show that NFATc4 protein is expressed in metaplastic areas during inflammation-induced pancreatic carcinogenesis using mouse models and human tissues. Importantly, genetic or pharmacological inactivation of NFATc4 abrogates transcriptional activation of* Sox9* and hinders development of metaplastic pancreatic lesions.

## 2. Materials and Methods

### 2.1. Animals

Generation and characterization of* pdx1-Cre* and* LSL-Kras* ^*G12D*^ animals have been described previously [2, 19]. Mouse strains were interbred to obtain* Kras* ^*G12D*^;* pdx1-Cre* animals. All strains had a C57Bl/6 background. For genotyping, PCRs were performed following digestion of tail cuts by using PCR buffer with nonionic detergents (PBND) and protein kinase (Applichem, Darmstadt, Germany). For induction of chronic pancreatitis, 8-week-old mice were subjected to caerulein (50 *μ*g/kg, 3 times/week) or dimethyl sulfoxide (DMSO) treatment for 4 weeks. All animal experiments were performed using protocols approved by the Institutional Animal Care and Use Committee at the Philipps University Marburg in Germany.

### 2.2. Acinar Cell Isolation

Acinar cell isolation was performed as described previously [17]. Briefly, the whole pancreas was dissected and incubated at 37°C in a collagenase I-containing solution. The minced pancreas was passed through a 105 *μ*m nylon filter to isolate acinar cells. After repetitive washing steps acinar cells were exposed to culture solution containing 30% fetal calf serum for a short time. Then cells were cultured in collagen supplemented with 1% heat-inactivated fetal bovine serum and, unless indicated otherwise, treated with DMSO and/or ddH_2_O, CsA (1 *μ*M), TGF*α* (50 ng/mL), or EGF (20 ng/mL). Ducts were counted after indicated time points using brightfield microscopy and Hoechst 33324 staining to show cell viability.

### 2.3. Cell Lines and Transfections

The acinar cell line 266-6 was obtained from Jemal et al. [20]. Primary tumor cells from* Kras* ^*G12D*^; *p*53^Δ/*wt*^;* pdx1-Cre* and* Kras* ^*G12D*^; *p*53^Δ/*wt*^;* EGFR* ^−/−^;* pdx1-Cre* mice were a kind gift from Jens Siveke, TU Munich, Germany. Cells were cultured in Dulbecco's modified Eagle medium (DMEM, Gibco, Darmstadt, Germany) supplemented with 10% fetal calf serum (Gibco) or in serum-containing DMEM supplemented with 1% nonessential amino acids, respectively. For EGF and TGF*β* treatment, cells were starved in serum-free medium for 24 h and afterwards stimulated with EGF (20 ng/mL) or with TGF*β* (10 ng/mL) as indicated.

For NFATc4 shRNA delivery in acinar cell explants, NFATc4 shRNAs (#1 5′-CGAGGTGGAGTCTGAACTTAA-3′; #2 5′-GCCAGACTCTAAAGTGGTGTT-3′) or control shRNAs (5′-CCTAAGGTTAAGTCGCCCTCG-3′) were infected using a lentiviral infection system as previously described [21]. For transfection of 266-6 acinar cells, NFATc4 siRNA was obtained from Life Technologies (5′-ccaguccaggucuacuuuutt-3′). Cells were transfected with NFATc4 siRNA for 48 h, using lipofectamine 2000 (Invitrogen). The constitutive active EGFR construct was obtained from Martin Privalsky. For reexpression of constitutive active EGFR, cells were transfected with either 3 *μ*g of EGFR or a control vector utilizing lipofectamine 2000 (Invitrogen).

### 2.4. RNA Isolation and Quantitative Real-Time PCR Analysis

For RNA isolation from acinar cells, solidified collagen was dissolved using collagenase I (Sigma). RNA from cell lines and acinar cells was isolated utilizing the RNeasy Mini Kit (Qiagen, Hilden, Germany) and first-strand complementary DNA was synthesized from 1 *μ*g of total RNA using random primers and the Omniscript Reverse Transcriptase Kit (Qiagen). RPLP0 was used as a housekeeping gene for normalization of gene expression. Mouse specific primers with the following sequences were used for expression analysis: NFATc1 (forward: 5′-tgggagatggaagcaaagac-3′; reverse: 5′-atagaaactgacttggacggg-3′), NFATc2 (forward: 5′-aagaggaaacgaagtcagcc-3′ reverse: 5′-tgggtgctgtgggtaatatg-3′) NFATc3 (forward: 5′-gaaactgaaggtagccgagg-3′ reverse: 5′-ctggtaaaatgcatgaggtcg-3′), NFATc4 (forward: 5′-tcttcaggacctctgcccta-3′; reverse: 5′-agcctaggagcttgaccaca-3′), Sox9 (forward: 5′-cgtgcagcacaagaaagacca-3′; reverse: 5′-gcagcgccttgaagatagcat-3′), cytokeratin 19 (forward: 5′-cctcccgcgattacaaccact-3′; reverse: 5′-ggcgagcattgtcaatctgt-3′), cytokeratin 6 (forward: 5′-gagcagatcaagaccctcaac-3′; reverse: 5′-aaacataggctccaggttctg-3′), EGFR (forward: 5′-acactgctggtgttgctgac; reverse: 5′-cccaaggaccacttcacagt-3′), and RPLP0 (forward: 5′-tgacatcgtctttaaaccccg-3′; reverse: 5′-tgtctgctcccacaatgaag-3′).

### 2.5. Protein Isolation and Immunoblotting

For protein isolation from cell lines, whole cell lysates were prepared using lysis buffer (50 mmol/L HEPES, pH 7.5–7.9, 150 mmol/L NaCl, 1 mmol/L ethylene glycol-bis(*β*-aminoethylether)-N,N,N′,N′-tetraacetic acid, 10% glycerin, 1% Triton X-100, 100 mmol/L NaF, and 10 mmol/L Na_4_P_2_O_7_ × 10 H_2_O) containing protease inhibitors. For immunoblot analysis 20 *μ*g of protein extracts was electrophoresed through 10% sodium dodecyl sulfate/polyacrylamide gels and transferred onto nitrocellulose membranes (Millipore, Darmstadt, Germany). Protein detection was performed using the following antibodies: NFATc4 (Abcam, ab62613, 1 : 1000) and *β*-actin (Sigma, 1 : 20000).

### 2.6. Histological Evaluation and Immunohistochemistry

Human chronic pancreatitis samples were obtained from 9 different patients and were derived from the Institute of Pathology of the Philipps University Marburg in accordance with the ethical regulations of the institute. For immunohistochemistry, formalin-fixed, paraffin-embedded tissue was sectioned (4 *μ*m). H&E staining and immunohistochemistry were performed as described previously [22]. The following antibodies were used: NFATc4 (Santa Cruz, sc13036), EGFR (Abcam, ab52894), pEGFR (Santa Cruz, sc101668), and Sox9 (Abcam, ab26414).

### 2.7. Chromatin Immunoprecipitation (ChIP)

ChIP analysis in 266-6 cells was performed as described previously [23]. Briefly, cells were cross-linked in medium containing 1% formaldehyde for 10 minutes at room temperature. Cross-linking was terminated utilizing 2.5 mol/L glycine. Cells were washed and harvested in ice-cold PBS. Nuclear lysates were obtained and DNA was sheered to fragments of 500 base pairs by sonication. The following antibodies were added to precleared chromatin for overnight incubation at 4°C: NFATc4 (Abcam, ab62613, 4 *μ*g), H3K4me3 (Cell Signalling 9727s, 2 *μ*g), RNA-polymerase II (Millipore, 05-623, 2 *μ*g), rabbit IgG (Santa Cruz, sc-2027, 2 *μ*g), and mouse IgG (Santa Cruz, sc-2025, 2 *μ*g). Protein A or G agarose was added and incubated for 2 hours. Beads were washed as described before [23] and reversion was performed using RNAse A (R4642, Sigma), protein kinase, and 5 mol/L sodium dodecyl sulfate overnight at 65°C. DNA was isolated using a phenol-chloroform protocol and analyzed via qRT-PCR utilizing Sox9 primers with the following sequences: +370 (forward: 5′-cgcgtatgaatctcctggac-3′, reverse: 5′-ggtgttctccgtgtccg-3′) and −825 (forward: 5′-ccgggaaaggacttgtcag-3′, reverse: 5′-tctggttcaacgaagctgg-3′).

## 3. Results

### 3.1. NFATc4 Is Highly Induced during EGFR-Mediated Acinar-to-Ductal Metaplasia

**Figure 1:**
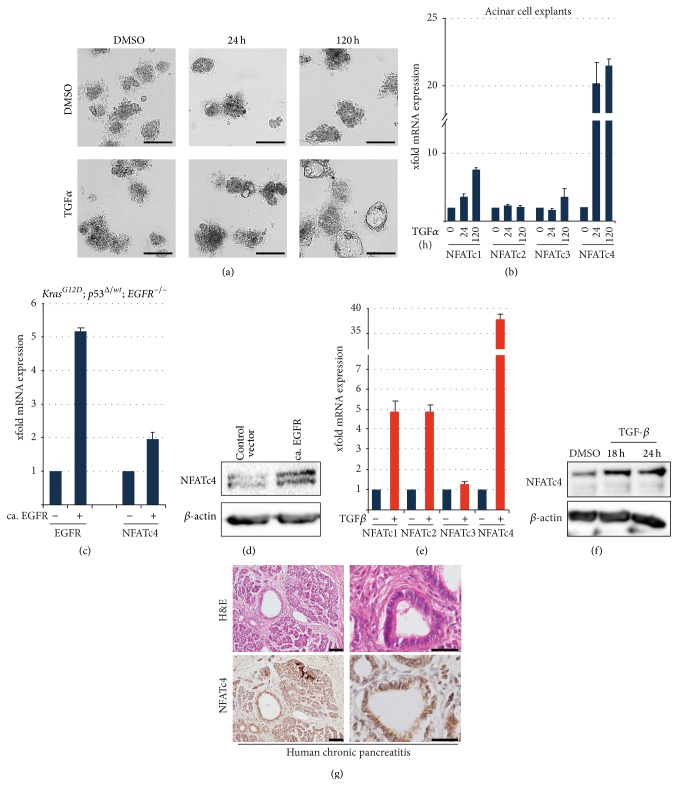
NFATc4 is induced in acinar cell transdifferentiation. (a) Light microscopy shows morphology of acinar cell extracts from wildtype mice in response to TGF*α* for the indicated time points (scale bar represents 100 *μ*m). (b) qRT-PCR to detect mRNA expression of NFAT isoforms in transdifferentiating acinar cells of wildtype mice in response to TGF*α*. (c)-(d) NFATc4 mRNA (c) and protein (d) expression levels in* Kras* ^*G12D*^; *p*53^Δ/*wt*^;* EGFR* ^−/−^;* pdx1-Cre* mice were determined after transient overexpression of a control vector or constitutive EGFR, respectively. NFATc4 mRNA expression following EGFR overexpression was normalized to control (means ± SD). (e) qRT-PCR reveals mRNA expression of NFAT isoforms in primary tumor cells from* Kras* ^*G12D*^; *p*53^Δ/*wt*^;* pdx1-Cre* mice following TGF*β* treatment for 24 hours. (f) Western Blot analysis in primary tumor cells from* Kras* ^*G12D*^; *p*53^Δ/*wt*^;* pdx1-Cre* mice upon TGF*β* treatment. *β*-actin was utilized as a loading control. (g) Representative H&E staining and immunohistochemistry were performed in human chronic pancreatitis samples. Scale bar represents 100 *μ*m. Asterisks indicate NFATc4 positive nuclei.

In addition to its pivotal role in PDAC initiation, acinar-to-ductal metaplasia represents a cellular adaptation process of the acinar cell in response to external stress signals which prevents stress-mediated cell death and can result in a stepwise regeneration program that reconstitutes pancreatic tissue integrity [24–26]. Either acute or chronic pancreatic inflammation results in the formation of metaplastic pancreatic lesions that can redifferentiate into acinar cells in a permissive cellular context [27]. Metaplastic lesions of chronic pancreatitis patients regularly express high levels of the epidermal growth factor receptor (EGFR) family and their natural ligands EGF and TGF*α* [12, 13, 16]. Recent reports have indicated that EGFR activation integrates external inflammatory cues into intracellular programs that mediate acinar cell adaptation and dedifferentiation [9, 17, 18, 28]. Accordingly, in an* in vitro* approach utilizing acinar cell explants from wildtype mice, activation of EGFR signaling by stimulation with TGF*α* strongly promoted transdifferentiation of explanted acinar cells into cells with duct-like characteristics (Figure 1(a)). The striking transdifferentiation behavior of pancreatic acinar cells in response to EGFR signaling activation is based on stepwise dedifferentiation processes of acinar cells and subsequent activation of ductal initiation programs [9, 18]. This crucial regulation of cellular plasticity is at least partially mediated by spatially and temporally controlled activation of the nuclear factor of activated T-cell (NFAT) family of transcription factors [22]. NFAT is a critical mediator of Ca^2+^/calcineurin signaling. NFAT functions were initially recognized in T-cell activation, where it regulates vital cellular functions related to adaptation and differentiation [29]. Importantly, expression and function of NFAT proteins are by no means limited to immune cells. Rather, cellular adaptation in several tissues has been attributed to NFAT-dependent signaling and transcription [30, 31]. In pancreatic acinar cells, basal expression levels of the four Ca^2+^/calcineurin-responsive NFAT family members were low (Figure 1(b)). Strikingly, TGF*α* stimulation of the acinar cell explants resulted in a time-dependent rise of NFATc1 and NFATc4 mRNA levels, while the expression of NFATc2 and NFATc3 was not altered (Figure 1(b)), consistent with previous reports suggesting that NFAT proteins do not have redundant functions [32]. The most prominent response to TGF*α* treatment was observed for NFATc4, suggesting that activation of this particular isoform might be essential during EGFR signaling-dependent acinar-to-ductal transdifferentiation (Figure 1(b)). To test whether EGFR signaling is required for NFATc4 activation, we utilized primary PDAC cells from* Kras* ^*G12D*^; *p*53^Δ/*wt*^;* EGFR* ^−/−^;* pdx1-Cre* mice with a homozygous deletion of EGFR. As shown in Figures 1(c) and 1(d), NFATc4 expression is low in EGFR depleted cells but is inducible in response to transient overexpression of constitutive active EGFR (Figures 1(c) and 1(d)). However, the induction of cellular plasticity in pancreatic exocrine cells has also been reported following treatment with the inflammatory cytokine TGF*β* [33] and TGF*β* treatment of pancreatic tumor cells promotes a transdifferentiation program leading to epithelial to mesenchymal transition (EMT) [33]. Most strikingly, TGF*β* stimulation of primary PDAC cells obtained from* Kras* ^*G12D*^; *p*53^Δ/*wt*^;* pdx1-Cre* mice resulted in the induction of several NFAT genes, including robust induction of NFATc4 mRNA and protein (Figures 1(e) and 1(f)). Lastly, we observed increased NFATc4 staining in the nuclei of cells within the metaplastic areas of a tissue from a patient with chronic pancreatitis (Figure 1(g)). Taken together, these data are consistent with the notion that NFATc4 is induced during the process of ADM in a mouse explant model and in human pancreatic tissue.

### 3.2. NFATc4 Is Involved in EGFR-Mediated Acinar-to-Ductal Metaplasia

**Figure 2:**
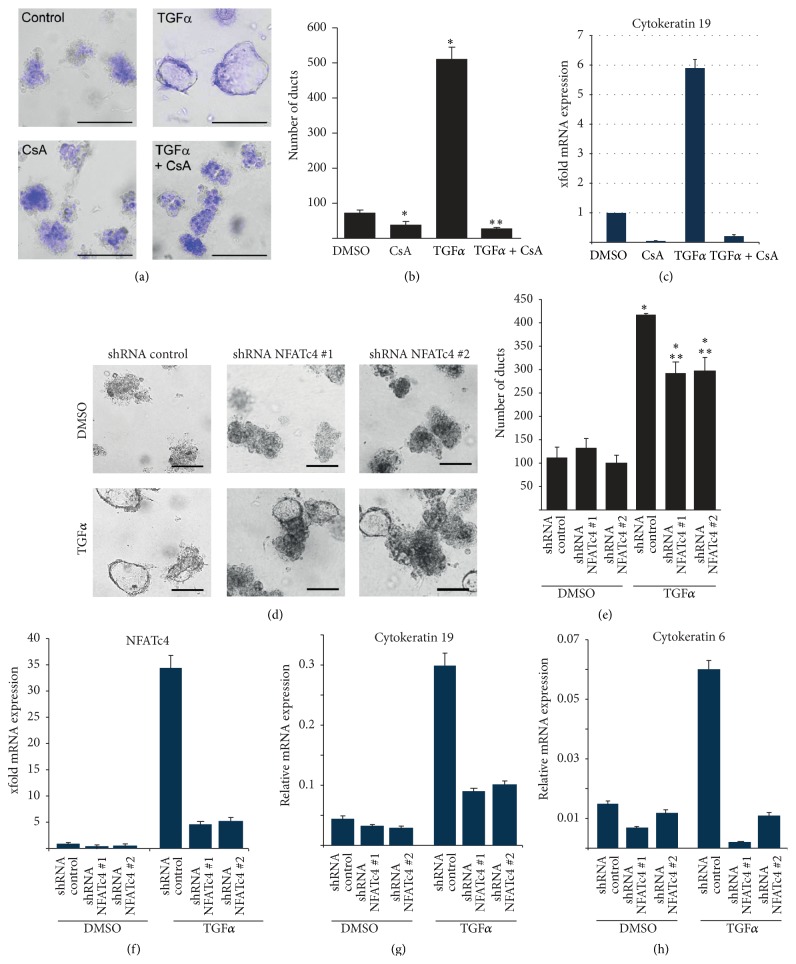
NFATc4 is required for acinar-to-ductal transdifferentiation in the pancreas. (a) Brightfield microscopy reveals acinar cell morphology upon TGF*α* and/or CsA treatment for 5 days (scale bars indicate 100 *μ*m). Hoechst 33342 was used to stain alive cells embedded in collagen at day 5. (b) Quantification of duct formation was performed five days after acinar cell extraction from wildtype mice. (c) qRT-PCR shows cytokeratin 19 expression in acinar cell explants upon indicated treatments for 5 days. (d)–(h) Acinar cells from wildtype mice were subjected to two different NFATc4 shRNAs or shRNA controls and were stimulated with TGF*α* for 5 days. (d) Brightfield microscopy was conducted to determine acinar cell morphology (scale bars indicate 100 *μ*m). (e) Quantification of duct formation was performed on day 5 following acinar cell extraction. (f)–(h) mRNA expression levels of the indicated genes were detected on day 5 following acinar cell extraction.

To test whether induction of NFATc4 is crucial for acinar-to-ductal conversion, we cotreated acinar cell explants with TGF*α* and cyclosporin A (CsA), a clinically used calcineurin inhibitor which prevents NFAT activation. Inhibition of calcineurin-NFAT signaling significantly attenuated acinar-to-ductal conversion, even in the presence of EGFR activation (Figure 2(a)). Consequently, duct formation was inhibited and cytokeratin 19 expression was decreased following CsA treatment (Figures 2(b) and 2(c)). Since pharmacological inhibition of calcineurin neither specifically inhibits the NFAT signaling pathway nor targets individual members of the NFAT family, we extended our analysis and genetically depleted NFATc4 in acinar cell explants using two specific NFATc4 shRNAs (Figure 2(d)). Importantly, depletion of NFATc4 expression significantly reduced TGF*α*-mediated duct formation in this ADM assay (Figures 2(d) and 2(e)). Consistent with the reduced duct-forming capacity of NFATc4-depleted acinar explants, we found diminished expression of cytokeratins 6 and 19, two marker genes that are induced during the process of acinar-to-ductal differentiation (Figures 2(f)–2(h)). Taken together, these data implicate NFATc4 as a critical EGFR-induced transcription factor involved in acinar cell reprogramming.

### 3.3. NFATc4 Is Induced during Pancreatic Cancer Initiation

**Figure 3:**
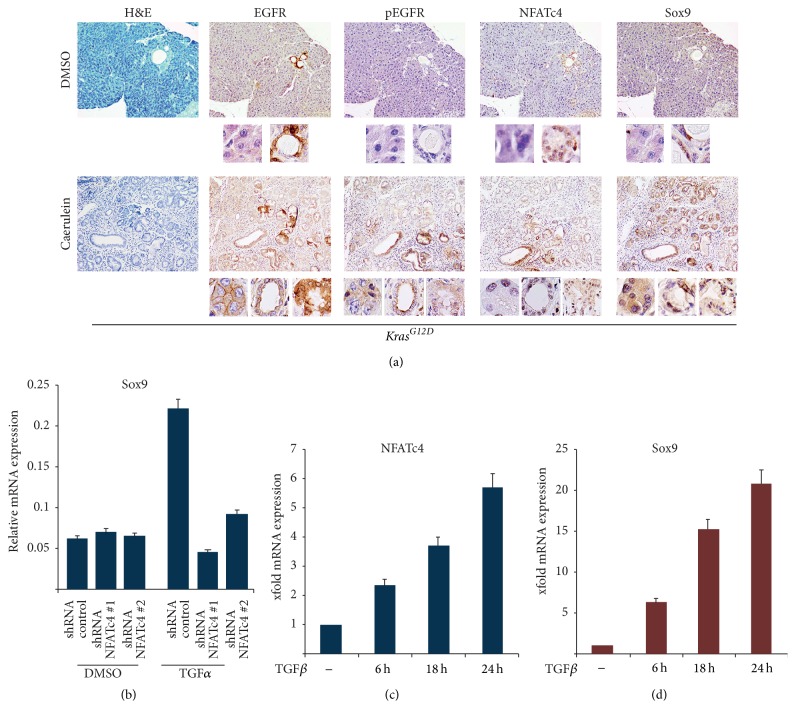
NFATc4 activation in metaplastic cells is associated with transcriptional induction of* Sox9*. (a) H&E staining as well as immunohistochemistry in 3-month-old* Kras* ^*G12D*^;* pdx1-Cre* mice following a four-week spanning treatment with caerulein or DMSO. The black boxes beyond the immunohistochemical staining display representative magnifications of normal acinar cells (left boxes), ADM (right box for the control group, middle box for the caerulein-treated mice), and PanIN lesions (right boxes in the caerulein cohort). (b) qRT-PCR reveals Sox9 mRNA expression in acinar cell explants from wildtype mice following genetic depletion of NFATc4 and TGF*α* stimulation for 5 days. (c)-(d) Primary pancreatic tumor cells from* Kras* ^*G12D*^; *p*53^Δ/*wt*^;* pdx1-Cre* mice were treated with TGF*β* for the indicated time points. mRNA expression of NFATc4 (c) and Sox9 (d) was determined by using qRT-PCR.

Acinar-to-ductal transdifferentiation is not limited to the physiological mechanisms of cellular adaptation, but it is now appreciated for its pivotal role in PDAC initiation [8]. Importantly, progression of inflammation-driven ADM to neoplastic lesions requires oncogenic mutation of* Kras* (*Kras* ^*G12D*^) in the metaplastic epithelial cell to block acinar redifferentiation and reconstitution of normal pancreatic architecture [34]. Hence, inflammation-induced EGFR signaling cooperates with oncogenic* Kras* mutation to promote pancreatic carcinogenesis [9, 22]. To examine whether NFATc4 activation is involved in PDAC initiation, we took advantage of an* in vivo* model of inflammation-driven pancreatic carcinogenesis. Specifically, we utilized a genetically engineered mouse model (GEMM) harboring a pancreas specific* Kras* mutation (*Kras* ^*G12D*^;* pdx1-Cre* mice) and treated these animals with caerulein, a well-established inducer of pancreatic inflammation [22, 30, 34]. A 4-week treatment regimen led to severe exocrine pancreatic injury with disordered acinar structure and induction of acinar-to-ductal metaplasia and early PanIN lesions as a result of increased acinar cell plasticity and initiation of pancreatic carcinogenesis (Figure 3(a)). Importantly, EGFR expression was remarkably induced in metaplastic exocrine cells upon pancreatic inflammation, visualized by enhanced cytosolic localization of its active phosphorylated form in caerulein-treated mice (Figure 3(a)). As reported previously, the metaplastic areas were characterized by strong induction of nuclear Sox9 expression, a pivotal marker of cellular reprogramming (Figure 3(a)), while the control group displayed only low cytosolic expression of the transcription factor. Strikingly, metaplastic areas and PanIN lesions with high EGFR, pEGFR, and Sox9 expressions also showed a dramatic increase of NFATc4 expression (Figure 3(a)). Consistent with our analyses in human chronic pancreatitis samples, NFATc4 expression in ADM areas in response to caerulein administration was predominantly detected in the nucleus, thus indicating activity of the transcription factor.

### 3.4. EGFR Signaling Involves NFATc4-Dependent Transcriptional Activation of Sox9

The formation and maintenance of pancreatic metaplastic lesions depend on the expression and activation of transcription factors that drive acinar transdifferentiation toward a duct-like phenotype. Recently,* Sox9* was implicated in cellular plasticity upon PDAC initiation and progression [8, 22, 35]. In accordance with these observations, acinar cell explants showed an increase of Sox9 mRNA expression following EGFR activation (Figure 3(b)), suggesting that Sox9 expression during acinar cell conversion is regulated at the transcription level. The concomitant expression of NFATc4 and Sox9 in metaplastic pancreatic cells suggests a mechanistic connection of both transcription factors. This assumption is further strengthened by the fact that genetic depletion of NFATc4 expression not only alters duct formation in the ADM model but also abrogated expression of Sox9 mRNA (Figure 3(b)). Moreover, TGF*β*-mediated time-dependent induction of NFATc4 expression in primary tumor cells from* Kras* ^*G12D*^; *p*53^Δ/*wt*^;* pdx1-Cre* mice correlated with increased Sox9 expression (Figures 3(c) and 3(d)). Together, our data identify* Sox9* as an NFATc4 target gene and implicate that both transcription factors functionally cooperate to drive the transdifferentiation processes in the pancreas.

**Figure 4:**
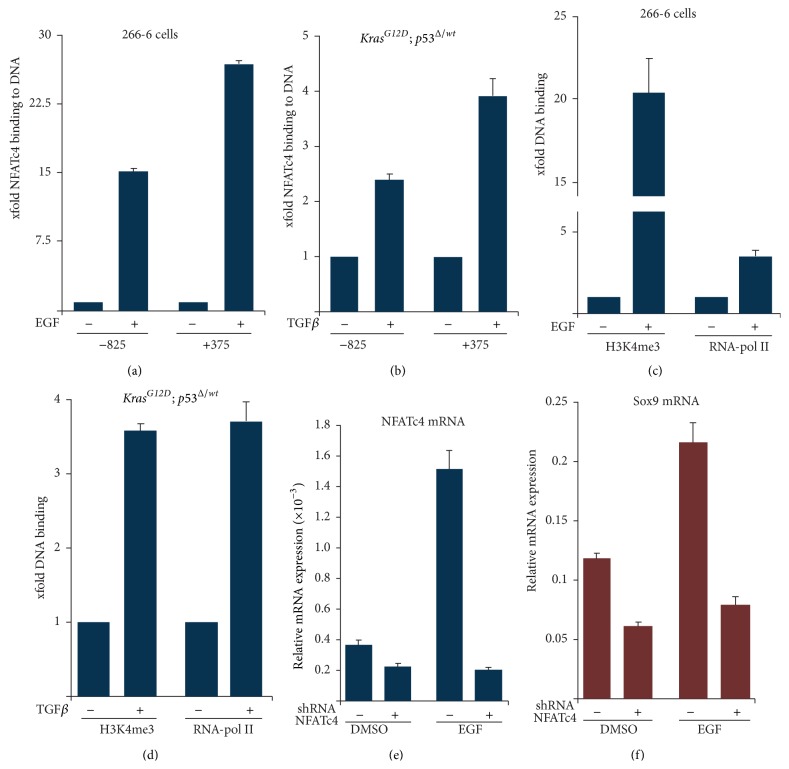
Sox9 represents a transcriptional NFATc4 target in metaplastic pancreatic cells. (a)–(d) ChIP analyses in 266-6 cells ((a) and (c)) and in primary tumor cells from* Kras* ^*G12D*^; *p*53^Δ/*wt*^;* pdx1-Cre* mice ((b) and (d)) were conducted in the following EGF (3 hours) or TGF*β* (24 hours) treatment, respectively. (a)-(b) qRT-PCR was performed to determine NFATc4 occupancy on the* Sox9* promoter regions −825 and +370. Data was normalized to IgG and displayed as xfold binding compared to control. (c)-(d) H4K3me3 and polymerase II enrichment are demonstrated on the Sox9 promoter region +370. Data was normalized to IgG and displayed as xfold binding compared to control. (e)-(f) qRT-PCR to detect NFATc4 (e) and Sox9 (f) mRNA expressions following genetic depletion of NFATc4 and EGF treatment (3 hours).

To further scrutinize the mechanistic correlation of NFATc4 and Sox9 expression, we performed chromatin immunoprecipitation (ChIP) analysis in the acinar cell line 266-6 upon activation of EGFR signaling and in primary tumor cells from* Kras* ^*G12D*^; *p*53^Δ/*wt*^;* pdx1-Cre* mice upon TGF*β* treatment. In both cell systems, EGFR activation as well as TGF*β* treatment resulted in an increased recruitment of NFATc4 to the* Sox9* promoter (Figures 4(a) and 4(b)), which correlated with enhanced occupancy of H3K4me3 and RNA-polymerase II on the* Sox9* promoter, indicating its transcriptional activity (Figures 4(c) and 4(d)). Both increased NFATc4 binding to the* Sox9* promoter and the enhanced promoter activity upon extracellular stimulation suggest a critical involvement of NFATc4 in the transcriptional regulation of Sox9. To verify this hypothesis, we genetically depleted NFATc4 expression in 266-6 acinar cells and investigated the impact of NFATc4 deficiency on EGFR-mediated Sox9 expression. Significantly, loss of NFATc4 significantly diminished EGFR-dependent induction of Sox9 expression in 266-6 acinar cells (Figures 4(e) and 4(f)).

Taken together, these data suggest that EGFR-stimulated NFATc4 gene expression and activation constitute a prerequisite for the transcriptional induction of* Sox9* and the process of acinar-to-ductal transdifferentiation.

## 4. Discussion

Acinar-to-ductal transdifferentiation represents a mechanism of cellular reprogramming in response to external stress signals, thus initiating pancreatic regeneration of damaged epithelial cells [36]. In the presence of an oncogenic Kras mutation, the function of ADM shifts from a preserver of pancreatic tissue integrity to a driver of neoplastic progression toward frank adenocarcinoma [34]. ADM induction and progression to neoplastic precursor lesions are closely linked to inflammation-induced activation of EGFR signaling [9, 17, 18]. Importantly, EGFR-mediated induction of ADM involves activation of genes that promote and maintain the ductal phenotype [37]. For instance, EGFR activation in response to epithelial injury has been shown to induce Sox9 expression in urothelial cancer [37], suggesting the involvement of an EGFR-Sox9 axis in malignant development and progression. The prominent function of Sox9 in tumorigenesis is also reflected in the pancreas, where Sox9 was described as a central transcription factor during acinar cell dedifferentiation and ductal conversion [8]. While loss of Sox9 during acinar cell dedifferentiation hindered ADM formation and neoplastic progression, ectopic induction of Sox9 accelerated Kras^G12D^-driven pancreatic carcinogenesis [8]. Although the relevance of an EGFR-Sox9 pathway has been demonstrated in several cellular contexts, the mechanisms linking inflammation-induced EGFR activation to Sox9 induction in PDAC initiation remained elusive. Here we identify the transcription factor NFATc4 as a functional link connecting inflammation-induced EGFR signaling to acinar metaplasia and show that induction of Sox9 in response to EGFR activation requires NFATc4-dependent transcriptional activation of the* Sox9* promoter.

The NFAT family of transcription factors has been reported to be involved in the integration of environmental signals into oncogenic processes that mediate proliferation, differentiation, and adaptation to inflammation in several cellular contexts [31, 38, 39]. For instance, recent work linked EGFR activation to NFAT-mediated Cox2 expression in colorectal tumorigenesis, thus pointing toward a critical role of the EGFR/NFAT cascade in the integration of inflammatory signals during carcinogenesis and progression [40]. In the pancreas, oncogenic activity has been demonstrated for the isoforms NFATc1 and NFATc2 that both accelerate Kras^G12D^-driven pancreatic carcinogenesis and PDAC progression in mice and humans [23, 31, 41, 42]. Importantly, the oncogenic activity of NFAT transcription factors not only is limited to neoplastic progression, but also comprises PDAC initiation. We recently showed that NFATc1 becomes activated in response to inflammation-induced EGFR signaling and complexes with c-Jun to promote acinar-ductal conversion by transcriptional induction of the* Sox9* gene [22]. Consistently, attenuation of NFATc1 activity using pharmacological or genetic approaches hindered EGFR-mediated promotion of acinar-to-ductal transdifferentiation [22], thus underlining the pivotal function of NFAT transcription factors in acinar cell adaptation and oncogenic conversion.

In comparison to its close relatives NFATc1 and NFATc2, less is known about the role of NFATc4 in pancreatic tissue adaptation and carcinogenesis. Here we show that NFATc4 is induced in an experimental setting of chronic pancreatitis and PDAC initiation and that inhibition of NFATc4 signaling preserves acinar cell morphology and function, indicating that the transcription factor might cooperate with oncogenic Kras signaling to promote pancreatic cancer initiation. The first evidence for a functional link between NFATc4 activation and neoplastic progression came from a study investigating patients with esophageal squamous dysplasia, which showed that enhanced expression levels of NFATc4 were associated with a higher chance of progression towards esophageal cancer [43]. Further underlining the role of NFATc4 in tumorigenesis, non-small lung cell cancer samples which were positively stained for oncogenic Cox2 displayed high expression levels of NFATc4 and the AP1 proteins c-Fos and c-Jun [44]. This is of particular interest, as c-Jun functions as the* bona fide* partner of NFAT transcription factors in pancreatic adaptation and neoplastic progression [22], suggesting that both transcription factors cooperate to drive acinar cell dedifferentiation.

In summary, we describe a novel mechanism of inflammation-induced cellular adaptation during PDAC initiation and propose a model in which NFATc4 links EGFR signaling activation to transcriptional induction of* Sox9* to promote acinar ductal dedifferentiation. Our data complements the wide functional spectrum of NFAT transcription factors in pancreatic adaptation and carcinogenesis and suggests that pharmacological strategies that target NFAT activation might pave the road for new preventive or therapeutic options in PDAC.

## 5. Conclusion

Pancreatic ductal adenocarcinoma (PDAC) represents a devastating disease with a dismal prognosis that has remained unchanged during the past several decades. Therefore, a better understanding of the molecular mechanisms that control PDAC initiation and progression is urgently needed in order to combat or prevent pancreatic cancer.

Herein we report a novel molecular mechanism that links EGFR signaling to activation of Sox9 during acinar-ductal metaplasia in pancreatic tissue adaptation and PDAC initiation. We identify the inflammatory transcription factor NFATc4 as a critical mediator of inflammation-induced EGFR signaling activation and demonstrate that NFATc4 drives acinar-to-ductal conversion and PDAC initiation through transcriptional induction of* Sox9*. Therefore, strategies designed to disrupt this pathway might be considered for the prevention and therapy of PDAC.
